# Hyperelastic Starch Hydrogel Configures Edible and Biodegradable All‐Components for Soft Robots

**DOI:** 10.1002/advs.202507216

**Published:** 2025-07-07

**Authors:** Siyu Yao, Haohao Hu, Mengfan Zhang, Qingqing Zhu, Donghong Liu, Shaoxing Qu, Guoyong Mao, Enbo Xu

**Affiliations:** ^1^ College of Biosystems Engineering and Food Science National Engineering Laboratory of Intelligent Food Technology and Equipment Zhejiang Key Laboratory for Agro‐Food Processing Fuli Institute of Food Science Zhejiang University Hangzhou 310058 China; ^2^ Innovation Center of Yangtze River Delta Zhejiang University Jiaxing 314102 China; ^3^ Department of Engineering Mechanics Zhejiang University Hangzhou 310027 China

**Keywords:** complete biodegradation, edible materials, flexible device, phase separation, stretchable starch chain

## Abstract

Developing edible and biodegradable structural materials is a promising solution to the increasing risk of plastic pollution. Starch has been widely used in foods such as noodles, and puddings for thousands of years, but with low mechanical performance. Here, a starch chain phase separation strategy is proposed in synthesizing starch‐based hydrogel to simultaneously enhance its strength and toughness, by the tunable interplay of glycerol/water (as ‐good solvent) and ethanol (as antisolvent). The mechanical performance of starch hydrogel, composed of starch, bound water, and glycerol, is widely tuned with maximum strains: 194.4–361.4%; maximum tensile stresses: 34–192 kPa; and Young's moduli: 36.0–205.8 kPa. Modulating the glycerol/ethanol ratio governs phase separation dynamics during the structural formation of starch hydrogel: lower glycerol/ethanol ratios bring higher maximum strain and maximum tensile stress, correlating with reconfigured starch crystallization and dynamic hydrogen‐bonding network. Notably, the hyperelastic starch hydrogel achieves complete soil degradation within 24 days and is constructed for a pneumatic soft gripper. This work pioneers a green and sustainable hydrogel platform that harmonizes high performance with edibility and biodegradability, offering transformative potential for eco‐friendly soft robotics and transient wearable systems.

## Graphical abstract

Hyperelastic starch hydrogel is tailored via a phase separation strategy of solvent‐antisolvent co‐modulation. The mechanical performance of starch hydrogel is widely tuned with maximum strains: 194.4–361.4%; maximum tensile stresses: 34–192 kPa; and Young's moduli: 36.0–205.8 kPa. Notably, the hydrogel achieves complete soil degradation within 24 days and is constructed for a pneumatic soft gripper demonstrating fine manipulation.

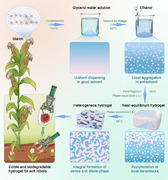

## 1 Introduction

It is projected that 8 million tons of solid waste will be generated worldwide every day by 2050.^[^ ^1^ ^]^ Elastomers of petroleum‐derived materials such as polyethylene terephthalate (PET),^[^ ^2^ ^]^ polypropylene (PP),^[^ ^3^ ^]^ polydimethylsiloxane (PDMS),^[^ ^4^ ^]^ thermoplastic polyurethane (TPU),^[^ ^5^ ^]^ poly(N‐isopropyl acrylamide) (PNIPAM),^[^ ^6^ ^]^ polyacrylic acid (PA),^[^ ^7^ ^]^ and synthetic rubber are extensively utilized in the fields of soft robotics,^[^ ^8^ ^]^ flexible electronics, and tissue engineering owing to their exceptional mechanical robustness and high toughness.^[^ ^9^ ^]^ Despite these advantages, their inherent non‐degradability and potential health risks are unfavorable for the human body,^[^ ^10^ ^]^ which challenges the feasibility of the green and sustainable material design principle. Hence, natural biobased and edible materials have emerged as a compelling solution due to their abundant sourcing, low‐cost processability, and complete biodegradability. They are ideal candidates for biocompatible conductive systems and flexible electronics, with the safety profile further enabling functional applications related to in‐vivo delivery robots^[^ ^11^ ^]^ and medical/food‐grade manipulating devices.^[^ ^12^ ^]^ However, the widespread adoption of edible materials in hydrogels is hindered by the characteristically inferior mechanical performance, including low stiffness, fracture strength, and toughness,^[^ ^13^ ^]^ which limits their functionality in practical scenarios. Consequently, it is a pivotal topic of tailoring the molecular and condensed structures of green, degradable, and even edible hydrogels with high‐performance mechanical properties.

Starch, as a biodegradable polysaccharide mainly derived from abundant plant sources,^[^ ^14^ ^]^ is widely utilized in food and non‐food industries. The versatile actions and natural advantages of starch position it as a promising candidate for sustainable materials: 1) low cost and broad availability; 2) easy biodegradation via environmental microbial or enzymatic attacking; 3) non‐toxicity, edibility, and high biocompatibility; 4) structural flexibility (often during gelatinization); and 5) product portability by lightweight fabrication.^[^ ^15^, ^16^ ^]^ Substantial studies have shown the potential of starches in fabricating functional materials, such as multi‐walled carbon nanotube‐reinforced starch films for conductivity,^[^ ^17^ ^]^ glassy carbon electrodes modified with graphene oxide, gold nanoparticles, and potato starch to detect environmental pollutants,^[^ ^18^ ^]^ and starch‐based hydrogel combined with polyacrylamide for human motion sensor and energy storage.^[^ ^19^ ^]^ However, these systems only use starch as a matrix (sometimes even less than half of the dry basis), and the functions rely on synthetic small molecules or polymer additives, compromising complete biodegradability and eco‐friendliness.

According to our knowledge, none of the starch‐based hydrogels balances robust mechanical properties (e.g., elastic modulus and fracture stress) with edible/degradable features.^[^ ^20^ ^]^ Moreover, the mechanical performance, such as stretchability and toughness at high‐starch levels, remains inadequate for applications. This gap stems from the complexity of starch's intricate multi‐scale structure, spanning glucose units, amylose/amylopectin chains, and their hierarchical assemblies.^[^ ^21^ ^]^ It is a big challenge in the elucidation of starch toughening mechanisms for precise molecular‐scale design and regulation. Therefore, we need a fundamental understanding of structure‐property relationships to enable targeted starch modifications and enhance its gel networks for sustainable, high‐performance starch materials.

General fabrication strategies of hydrogel systems such as double networks,^[^ ^22^ ^]^ nanocomposites,^[^ ^23^ ^]^ slip‐ring architectures,^[^ ^24^ ^]^ and ionically cross‐linked networks,^[^ ^25^ ^]^ primarily focus on cellular structure formation.^[^ ^26^ ^]^ In contrast, phase separation induces the hierarchical structures of hydrogels (across nanometer to millimeter scales) through precise compositional and processing adjustments.^[^ ^26^, ^27^ ^]^ It is a reconfiguration process of pre‐hydrogels driven by disparities in chemical or physical properties that induce immiscible components to segregate into distinct phases.^[^ ^28^ ^]^ Starch chains exhibit natural double‐helix crystallization,^[^ ^29^ ^]^ but their linear units also possess single‐helix “V‐type” crystalline conformations when absorbing guest molecules such as ethanol.^[^ ^21^, ^30^ ^]^ Ethanol acts as a poor solvent (or antisolvent) of starch to promote its aggregation and crystallization via unbalanced solute‐solvent‐antisolvent interactions.^[^ ^31^ ^]^ Herein, we proposed a phase‐separation approach of starch induced by the antisolvent of ethanol in a water/glycerol solvation medium, leveraging abundant hydroxyl groups on starch to facilitate the generation of dynamic hydrogen bonding with glycerol.^[^ ^20^ ^]^ By modulating the synergistic ethanol‐glycerol gradient, the phase separation kinetics and resultant micro/nanoscale architectures of starch chains were efficiently controlled to enhance mechanical properties, including elastic modulus, fracture stress, and toughness. Additionally, we constructed the prepared starch‐based hydrogel into a pneumatic soft gripper to demonstrate fine manipulation. Compared to PDMS grippers and other petroleum‐derived soft grippers, this starch‐based hydrogel gripper is safer, greener, and more sustainable when gripping soft and fragile food. At the end of their lifetimes, food‐grade grippers biodegrade and become nutrients for other organisms. In contrast, petroleum‐derived grippers become persistent waste and a source of pollution. Food‐grade grippers are therefore green, safe, and biodegradable, offering significant advantages for green manufacturing and sustainable development. They also have greater potential for use as an in‐vivo soft robot to deliver drugs and nutrients.^[^ ^32^ ^]^ This work achieved almost whole starch hydrogels coupled with high mechanical resilience for soft robotics as a demo, offering a sustainable pathway to develop edible, biodegradable, and hyperelastic materials.

## 2 Results and Discussion

### 2.1 Design and Preparation of Starch Hydrogels via Phase Separation

**Figure 1:**
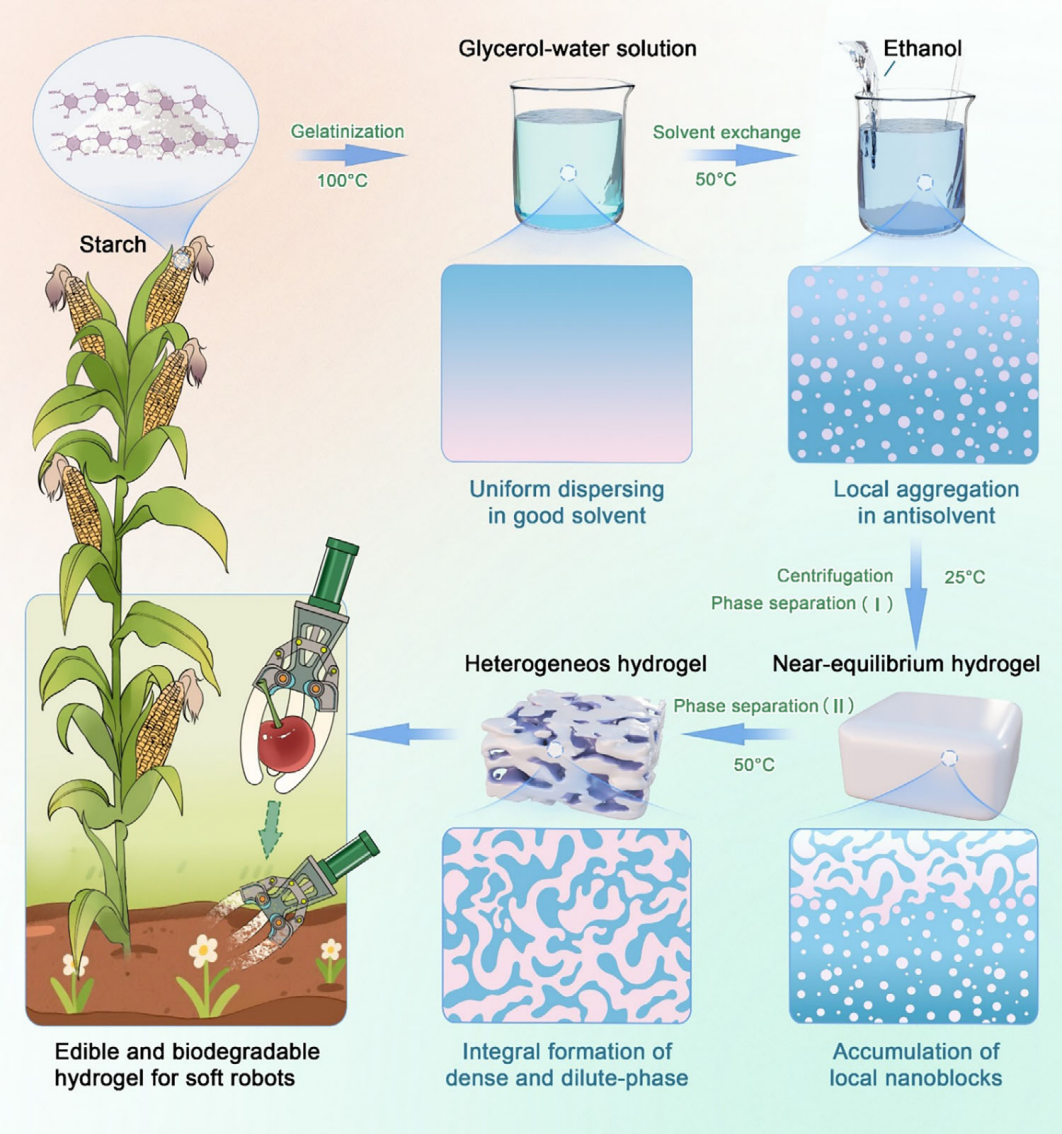
Schematic illustration of hyperelastic starch hydrogel preparation and its degradation in soil.

In this study, we presented a solvent‐regulated phase separation strategy to fabricate starch hydrogels with architecturally heterogeneous networks and enhanced mechanical performance. As depicted in **Figure** **1**, the synthesis process involved a two‐step solvent‐exchange protocol within a glycerol‐water dual‐solvent system for starch using ethanol as an antisolvent. Initially, native starch granules underwent thermal gelatinization at 100 °C in a glycerol‐water solution (i.e., good solvent), achieving near‐equilibrium network formation. Subsequently, controlled phase separation was triggered at 50 °C through progressive replacement with ethanol, inducing dual‐phase structural evolution: i) local aggregation of starch chains into dense sacrificial domains and ii) integral formation of interconnected dilute‐phase networks.^[^ ^33^ ^]^ This unique architecture was created with a stress‐redistribution mechanism, where the continuous dilute phase facilitated stress transfer while the dense domains acted as energy‐dissipating motifs through sacrificial bond rupture, synergistically enhancing the mechanical performance of the starch hydrogel.^[^ ^26^ ^]^ The detailed experimental design of starch hydrogels is shown in Table S1 (Supporting Information). The samples were named according to the glycerol and ethanol volume ratio used. For example, G0.4/E0.6 was 40% of glycerol aqueous solution, and the amount of ethanol was 0.6 times that of glycerol aqueous solution.

**Figure 2:**
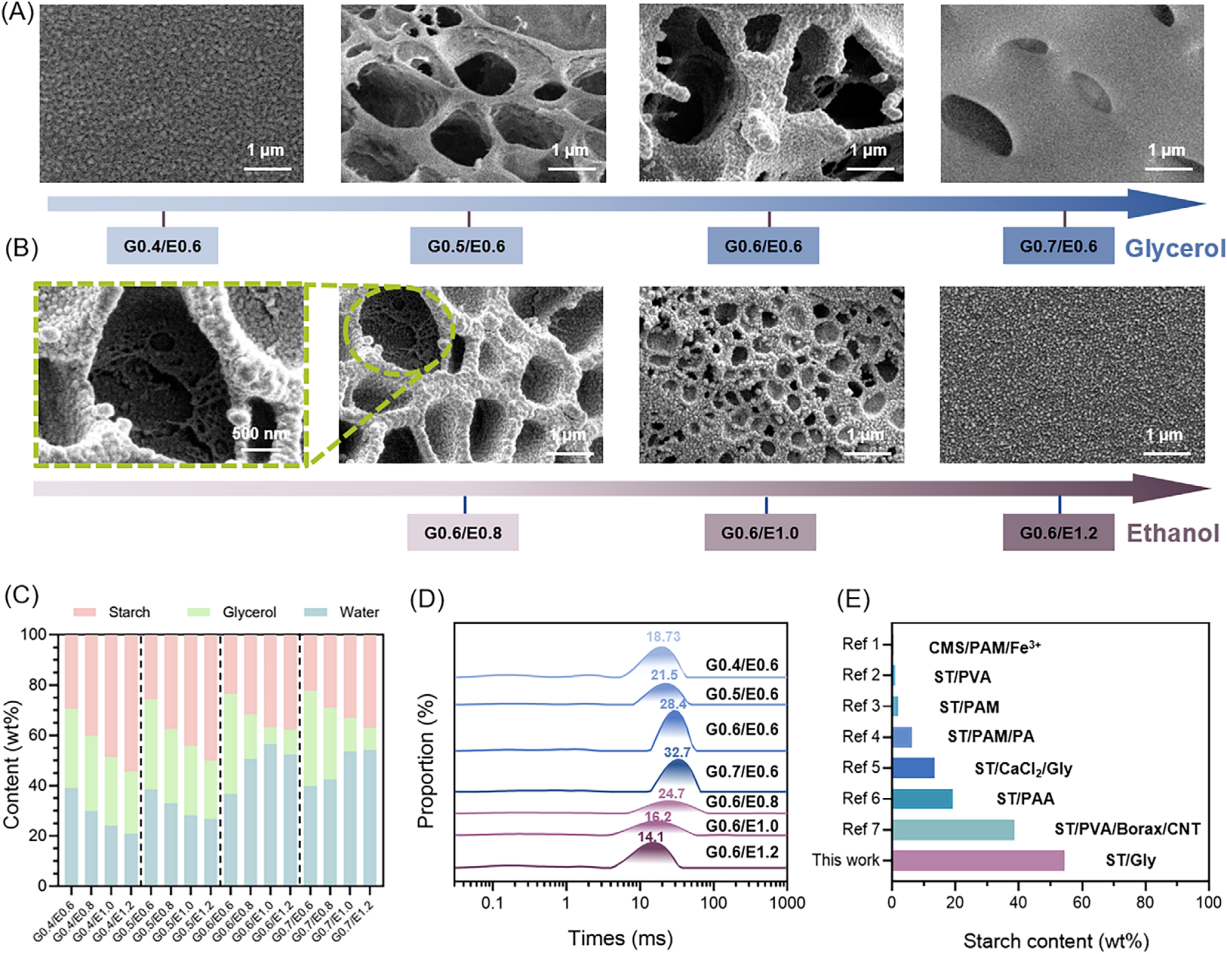
Tunable formation of multiscale microstructure inside the starch hydrogel. Cryo‐EM images of as‐prepared starch hydrogel of A) glycerol changed by set ethanol addition, with samples labeled as G0.4/E0.6, G0.5/E0.6, G0.6/E0.6, G0.7/E0.6, and B) ethanol changed by set glycerol addition, with samples labeled as G0.6/E0.8, G0.6/E1.0, G0.6/E1.2. C) The three main components of the hydrogel: are starch, glycerol, and water. D) Relaxation times of hydrogel samples in LF‐NMR. E) Summary of reported starch content (wt.%) in hydrogels. The data are extracted from the following references: this work (represented by G0.4/E1.2), (carboxymethyl starch/polyacrylamide/ferric ion, i.e., CMS/PAM/Fe^3+^),^[^ ^35^ ^]^ (starch/poly(vinyl alcohol), i.e., ST/PVA),^[^ ^36^ ^]^ (starch/polyacrylamide, ST/PAM),^[^ ^37^ ^]^ (starch/polyacrylamide/phytic acid, ST/PAM/PA),^[^ ^19^ ^]^ (starch/ calcium chloride /glycerol, ST/CaCl_2_/Gly),^[^ ^38^ ^]^ (starch/polyacrylic acid, ST/PAA),^[^ ^39^ ^]^ (starch/poly(vinyl alcohol)/borax/carbon nanotube, ST/PVA/Borax/CNT).^[^ ^40^ ^]^

The result of the Cryo‐electron microscopy (Cryo‐EM) test showed how solvent ratios dictated starch hierarchical structure (**Figure** **2**A,B). Nonporous topography dominated in the situation of low glycerol (e.g., G0.4/E0.6) or high ethanol (e.g., G0.6/E1.2), which seemingly consisted of dense starch domains (similar to inter‐cross‐linked nanoblocks) with a homogenous distribution. Increasing glycerol or decreasing ethanol proportions induced the appearance of arc‐shaped pores (based on substantial nanoblocks) with expanding sizes. For example, G0.6/E0.8 starch hydrogel exhibited the porous architectures formed by the aggregation of local nanoblocks, potentially due to an Ostwald ripening phenomenon during phase separation. It was speculated that after the local nucleation of starch chains, adjacent nucleating nanoaggregates will integrate into larger domains through polymer chain diffusion and polymer‐solvent interactions.^[^ ^34^ ^]^

The pore evolution correlated with the content and state of water in the hydrogel matrix as revealed by low‐field nuclear magnetic resonance (LF‐NMR) analysis. During the hydrogel formation process, excessive water and glycerol were primarily removed through centrifugation, while ethanol vanished via evaporation during the subsequent temperature‐controlled phase separation. As demonstrated in Figure 2C and Table S2 (Supporting Information), the starch‐based hydrogel compositions exhibited wide ranges of tunability: water (20.81–56.50 wt.%), starch (22.26–54.42 wt.%), and glycerol (6.71–39.84 wt.%). The increase of ethanol volume ratio improved starch proportion (sometimes dry basis > 80%) residual in the hydrogels. For instance, when ethanol initial content increased from 0.6 v_ethanol_/v_glycerol+water_ to 1.2 v_ethanol_/v_glycerol+water_, final proportions of the starch increased from 29.35 to 54.42 wt.% with glycerol fixed at G0.4. Ethanol also suppressed residual retention of glycerol. For example, compared with G0.7/E0.6, the final glycerol proportion of G0.7/E1.2 decreased by 4.30 times. Increasing the initial amount of glycerol promoted the water fixation of starch hydrogels (such as those prepared from G0.4/E1.2 to G0.7/E1.2 with water proportion from 20.81 to 54.16 wt.%). The compositional alternations were governed by solvent‐polymer affinity during phase separation: ethanol‐induced dehydration preferentially consolidated starch networks, while glycerol acted as a hygroscopic agent to stabilize bound water states.

All starch hydrogel samples exhibited bound water signatures in LF‐NMR curves (relaxation times < 40 ms, Figure 2D). When the initial glycerol content increased, the relaxation time of the hydrogels increased from 18.73 ms (G0.4/E0.6) to 32.7 ms (G0.7/E0.6); when the initial ethanol increased, the relaxation time of the hydrogels decreased from 28.4 ms (G0.6/E0.6) to 14.1 ms (G0.6/E1.2). It indicated that the rise of the glycerol/ethanol ratio elongated relaxation times, weakening water‐starch matrix interactions. The looser the bound water, the stronger the sublimation of water within equal freezing time. It resulted in larger pores, which was consistent with the Cryo‐EM results. The starch hydrogels prepared by the phase separation method were edible with starch, glycerol, and bound water. For example, the G0.4/E1.2 hydrogel containing 54.42 wt.% starch, 24.77% glycerol and 20.81 wt.% water were safer to eat and use compared with other starch‐based hydrogels, such as carboxymethyl starch/polyacrylamide/ferric ion (CMS/PAM/Fe^3+^),^[^ ^35^ ^]^ starch/poly(vinyl alcohol) (ST/PVA),^[^ ^36^ ^]^ starch/polyacrylamide (ST/PAM),^[^ ^37^ ^]^ starch/polyacrylamide/phytic acid (ST/PAM/PA),^[^ ^19^ ^]^ starch/calcium chloride/glycerol (ST/CaCl_2_/Gly),^[^ ^38^ ^]^ starch/polyacrylic acid (ST/PAA),^[^ ^39^ ^]^ starch/ poly(vinyl alcohol)/borax/carbon nanotube (ST/PVA/Borax/CNT) (Figure 2E; Table S3, Supporting Information).^[^ ^40^ ^]^

### 2.2 Molecular Structure of Rearranged Starch Chains in Hydrogel

**Figure 3:**
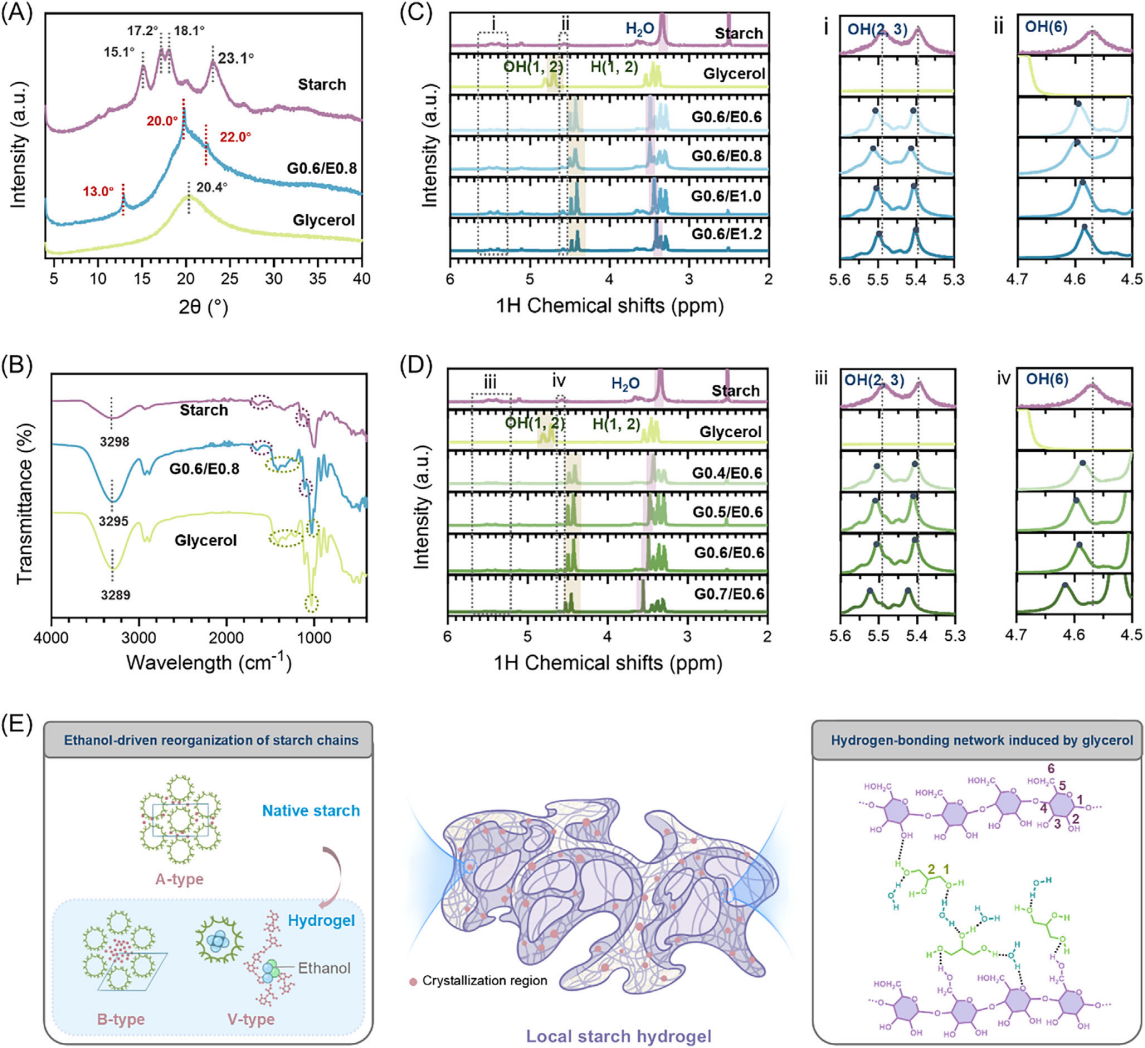
Molecular and condensed structures of starch hydrogel. A) Crystalline properties of native starch, glycerol, and as‐prepared hydrogel (represented by G0.6/E0.8) in XRD patterns. B) Hydrogen‐bonding cross‐linking performed by FTIR spectra. C) 1H NMR of native starch, glycerol, and hydrogels controlled in ethanol gradient (G0.6/E0.6, G0.6/E0.8, G0.6/E1.0, G0.6/E1.2), with magnified peaks at (i) 5.6–5.3 ppm and (ii) 4.7–4.5 ppm. D) 1H NMR of hydrogels controlled in glycerol gradient (G0.4/E0.8, G0.5/E0.8, G0.6/E0.8, G0.7/E0.8), with magnified peaks at (iii) 5.6–5.3 ppm and (iv) 4.7–4.5 ppm. E) Ethanol‐driven crystallization, transformation, and hydrogen‐bonding network induced by glycerol in starch hydrogels.

Ethanol‐driven phase separation induced supramolecular reorganization of starch chains against glycerol, to trigger chain entanglement and densification. X‐ray diffraction (XRD) analysis revealed a solvent‐mediated crystalline polymorphism of starch hydrogel (**Figure** **3**A; Figure S1, Supporting Information). Native starch exhibited characteristic A‐type crystal (15.1°, 17.2°, 18.1°, 23.1°), while phase‐separated starch hydrogels developed hybrid B (22°)+V (13°, 20.0°)‐type crystals, respectively.^[^ ^21^ ^]^ This transition of condensed structure might be related to the disruption of native double helices and subsequent reorganization of starch chains into metastable single and double‐helix‐dominant aggregates at the nano/microscale. It was accompanied by a spatial rearrangement of starch chain packing.

We designed a control group without ethanol addition (G0.6/E0) by gelatinizing starch in a glycerol‐water solution, subjected to the same equilibration conditions, to examine how the phase separation process regulated the starch chains in the hydrogels. XRD analysis (Figure S2, Supporting Information) revealed dramatically attenuated crystallinity in G0.6/E0 compared to the phase‐separated hydrogel represented by G0.6/E0.8, confirming the critical role of ethanol in regulating the supramolecular order of starch chains. The double helices in the A‐type crystal of native starch were completely dissociated in solution, yielding amorphous chains with short‐range disorder; then, ethanol introduction disrupted solvent‐starch chain equilibrium, triggering chain realignment through competitive dehydration. This kinetic process promoted the simultaneous formations of V‐type single helices (ethanol‐stabilized hydrophobic cavities) and B‐type double helices (water molecules‐entrapped hydrophobic centers formed by hexagonally packed antiparallel starch double helices),^[^ ^41^ ^]^ ultimately establishing the main local dense stacking structures as energy‐dissipative domains.

Spectroscopic investigations elucidated the hydrogen‐bonding cross‐linking dynamics. FTIR (Figure 3B; Figure S3, Supporting Information) and 1H NMR (Figure 3C,D) analyses confirmed the absence of covalent bonding between starch, glycerol, and water. The O‐H stretching band in FTIR and differential NMR chemical shifts indicated the presence of 3D hydrogen‐bonding networks. FTIR spectrum of G0.6/E0.8 showed the characteristic peaks of glycerol (green dotted circle) and starch (purple dotted circle). A band at 3295 cm⁻¹ related to O‐H stretching was broadened and intensified, compared to the intensity of native starch (3298 cm⁻¹) and pure glycerol (3289 cm⁻¹). It might be attributed to the strengthened hydrogen‐bonding interactions within the ternary starch‐glycerol‐water network. The glycerol OH peak of hydrogels shifted to the right in the high‐field nuclear magnetic spectrum, indicating an increase in electron cloud density (khaki‐shaded regions in Figure 3C,D). However, both the peaks of OH groups in water (purple‐shaded regions in Figure 3C,D) and those in starch (Figure 3C i‐ii,D iii‐iv) shifted to the left, resulting in lower electron cloud density. This provided strong evidence for the generation of a cross‐linking network of the starch‐glycerol‐water system via hydrogen bonding. After the formation of hydrogels, the displacement of all OH functional groups changed in starch, glycerol, and water, indicating that they were hydrogen‐bonding cross‐linking sites with potential dynamic performance (Figure 3E).

When initial ethanol content increased (e.g., from G0.6/E0.6 to G0.6/E1.2, Figure 3C) or initial glycerol content decreased (e.g., from G0.7/E0.6 to G0.4/E0.6, Figure 3D), the peak shift of the hydrogels was closer to that of natural starch and pure glycerol. The larger the glycerol/ethanol ratio, the greater the chemical shift, showing an enhancement of hydrogen‐bonding cross‐linking within the molecules. This hydrogen‐bonding hierarchy enabled glycerol to act as a molecular bridge, synergistically hydrating starch matrices through multisite O‐H interactions while ethanol modulated phase separation kinetics. The dynamic equilibrium between solvent‐antisolvent competition (i.e., glycerol hydration vs ethanol dehydration, Figure S4, Supporting Information) and hydrogen‐bonding redistribution shaped hydrogel architecture, where higher glycerol/ethanol ratios stabilized strongly cross‐linked networks with enhanced water retention. Ethanol and glycerol synergistically modulate the conformational state of starch chains, thereby altering the free energy landscape of the system. Driven by hydrogen bonding, the starch structure transitions to a more stable hydrogel network state, achieving its lowest free energy configuration (Figure S5, Supporting Information).

### 2.3 Strength, Stiffness and Toughness of Hyperelastic Starch Hydrogel

**Figure 4:**
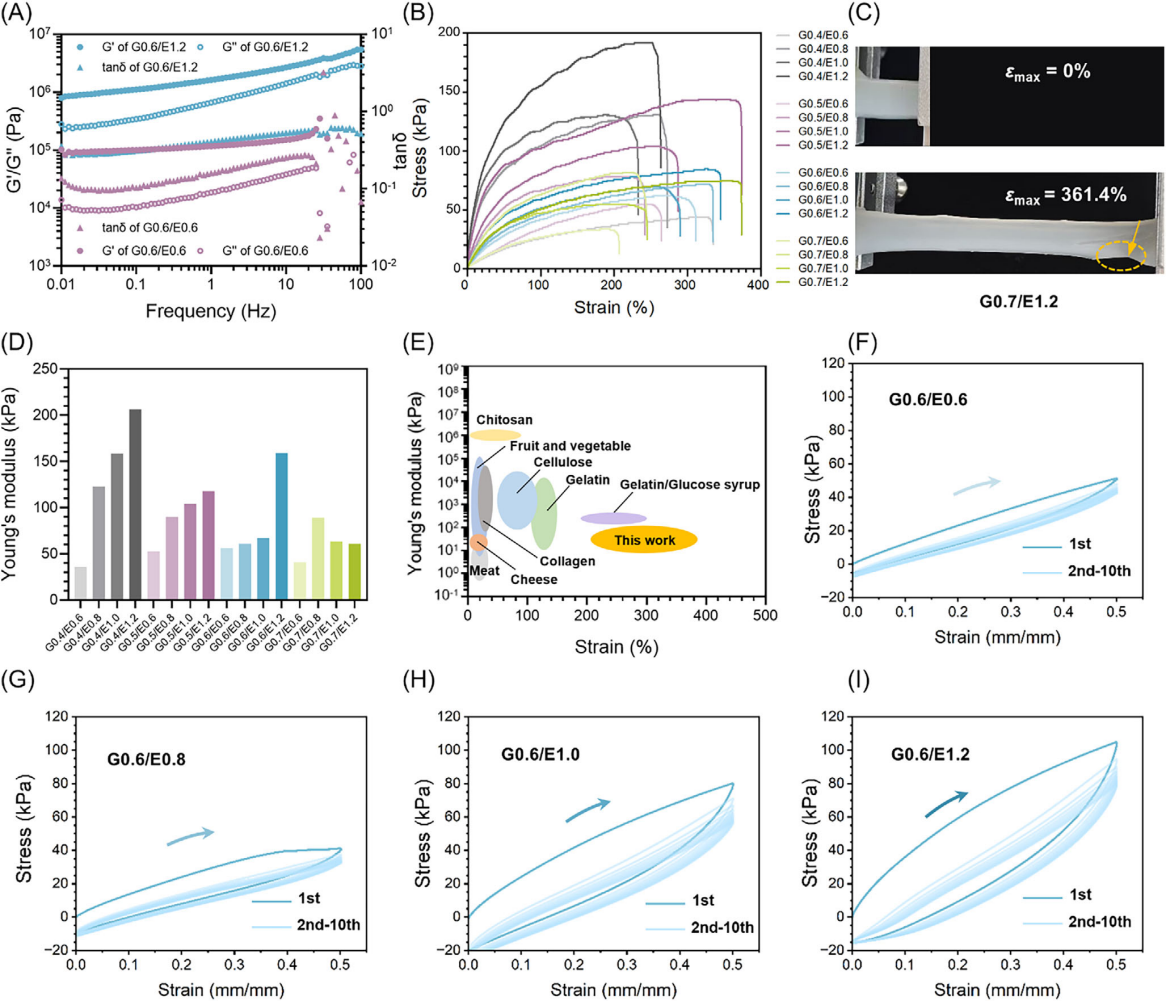
Mechanical performance of hyperelastic starch hydrogel. A) Frequency sweep curves of starch hydrogels at 25 °C. B) Tensile stress–strain curves of the starch hydrogels. C) Optical image of hyperelastic starch hydrogels (represented by G0.7/E1.2) after stretching. D) Young's modulus of starch hydrogels calculated by stress–strain curves. E) Comparison plots of starch hydrogels with reported edible hydrogels (based on meat cheese, collagen, cellulose, fruit and vegetable, chitosan, gelatin, and glucose syrup) by strain and Young's modulus, the data are extracted from the references.^[^ ^32^, ^45^ ^]^ Cyclic loading of F) G0.6/E0.6 hydrogel, G) G0.6/E0.8 hydrogel, H) G0.6/E1.0 hydrogel, I) G0.6/E1.2 hydrogel to a strain of 50%.

The rheological properties of starch hydrogels were identified by dynamic mechanical analysis (DMA). The storage modulus *G′* and loss modulus *G″* of the hydrogels are shown in **Figure** **4**A and S6 (Supporting Information). In the frequency range of 0.01–20 Hz set at 25 °C, *G′* was higher than *G″*, which proved that the starch hydrogel had a stable and highly cross‐linked network.^[^ ^19^, ^42^ ^]^ When scanning at high frequency (20–100 Hz), the chaotic values of G0.6/E0.6 indicated that the cross‐linked network underwent irreversible deformation under high stress, while the G0.6/E1.2 as hyperelastic starch hydrogel still maintained with stable values, which might be related to its enhanced toughness.

The mechanical performances of the starch hydrogels were systematically evaluated through uniaxial tensile testing. Stress–strain curves for starch hydrogels formulated with varying glycerol/ethanol ratios were shown in Figure 4B, with corresponding quantitative data provided in Table S4 (Supporting Information). The results revealed that both ethanol and glycerol acted as coordinated regulators of hydrogel mechanics. For instance, at fixed ethanol contents of 0.6, 0.8, and 1.0 v_ethanol_/v_glycerol+water_, respectively, incremental increases in initial glycerol content (40–70 wt.%) demonstrated minimal influence on maximum strain (*ε*_max_) but progressively reduced fracture tensile stress (*σ*_max_). The overall trend seemed that increasing the glycerol content would reduce stress and increase strain. Interestingly, when the initial ethanol content reached up to 1.2 v_ethanol_/v_glycerol+water_, contrasting alternation of mechanical behavior emerged. For instance, hydrogel G0.4/E1.2 exhibited *σ*_max_ = 192 kPa and *ε*_max_ = 252.1%, while G0.7/E1.2 showed a marked shift to *σ*_max_ = 75 kPa and *ε*_max_ = 361.4% (Figure 4C; Figure S7, Supporting Information). It might be due to the overhigh ethanol addition at relatively low glycerol inducing the intensification of phase separation. A distinct dual‐effect synergy was observed where the hyperelastic hydrogel showed both high maximum strain and high maximum stress by elevating ethanol addition, such as G0.5/E0.6 (*σ*_max_ = 55 kPa, *ε*_max_ = 252.3%) transitioned to G0.5/E1.2 (*σ*_max_ = 144 kPa, *ε*_max_ = 358.3%), and similarly, G0.7/E0.6 (*σ*_max_ = 34 kPa, *ε*_max_ = 194.4%) evolved to G0.7/E1.2 (*σ*_max_ = 75 kPa, *ε*_max_ = 361.4%, Figure 4C).

The systematic enhancement in both stress and strain under high‐level ethanol introduction suggested a possible mechanism based on the phase separation of starch substrate: ethanol‐induced chain aggregation likely 1) promoted increased cross‐linking density, which effectively restricted polymer chain slippage to elevate mechanical strength;^[^ ^19^ ^]^ 2) enhanced the effective chain length within the same distance, thereby amplifying macroscopic strain. Collectively, these findings established ethanol as a multifunctional modulator capable of tuning both the strength and toughness of hyperelastic starch hydrogels (Movies S1 and S2, Supporting Information).

Young's modulus, calculated from the linear elastic region of stress–strain curves (Figure 4D), showed a wide range of controllable elastic modulus values in starch hydrogels (from 30 to 210 kPa). The modulus exhibited an inverse correlation with glycerol content and a direct proportionality to ethanol additive amount, enabling synergistic control over network stiffness. Specifically, ethanol‐induced chain aggregation elevated modulus by reinforcing inter‐molecular interactions, and glycerol regulated the flexibility of materials by binding hydrogen bonding to water. Notably, these starch hydrogels achieved exceptional elastic deformability compared to other edible counterparts (e.g., those from chitosan, meat, cheese, fruit, and vegetable, Figure 4E), combining a lower modulus range (this work: 10^1^–10^3^ kPa vs fruit and vegetable‐derived materials: 10^1^–10^5^ kPa) with superior maximum tensile strain (this work: 200–400% vs fruit and vegetable‐derived materials: < 20%).^[^ ^32^ ^]^ Such tunable elasticity positions of starch hydrogels as promising candidates for flexible applications require compliance with biological tissues or dynamic mechanical responsiveness.

A continuous cyclic tensile test was conducted at a strain of 50% (Figure 4F–I and Figure S8, Supporting Information). The obvious hysteresis loops during loading and unloading indicated energy dissipation phenomena, which were related to the dynamic hydrogen‐bonding cross‐linking inside the starch‐glycerol‐water system. Initial loading–unloading cycles exhibited pronounced hysteresis loops, probably attributed to the rupture of transient hydrogen‐bonding cross‐links. Reduced hysteresis in subsequent cycles reflected the strain‐induced alignment of hydrogen‐bonding networks, which was beneficial for subsequent stretching and similar to previous studies.^[^ ^19^ ^]^ The loading–unloading curve and hysteresis region of the cycle matched well with each other, and the stress values were very similar. It demonstrated the rapid reconfiguration of reversible O‐H interactions in hyperelastic starch hydrogels, highlighting fatigue‐resistant self‐recovery (a hallmark of dynamically cross‐linked biopolymer networks). Notably, initial ethanol content almost decided the degree of dissipation capacity: at fixed ethanol content (1.0 v_ethanol_/v_glycerol+water_) for hydrogel fabrication, hysteresis areas remained consistent across glycerol contents (Figure S8, Supporting Information); while increasing the addition content of ethanol (e.g., from G0.6/E0.6 to G0.6/E1.2) amplified hysteresis (Figure 4F–I), which might be related to the ethanol‐controlled structure as previously described (Section 2.2). Excessive dehydration induced by ethanol promoted the densification and crystalline domain formation of starch chains. It also reduced relaxation time to restrict starch chain mobility.^[^ ^43^, ^44^ ^]^ Furthermore, from the perspective of structural dissipation, an increase in ethanol content resulted in smaller pores (not conducive to stress transfer) but more starch nano blocks as fundamental energy‐dissipating motifs. The hyperelastic starch hydrogels played a blueprint for designing sustainable materials with adaptive mechanical performance (Movie S3, Supporting Information), which was particularly suitable for the scenarios requiring cyclic load durability, such as soft gripper, as seen in Section 2.4.

### 2.4 Edible and Biodegradable All‐Components of Resilient Starch Hydrogel

**Figure 5:**
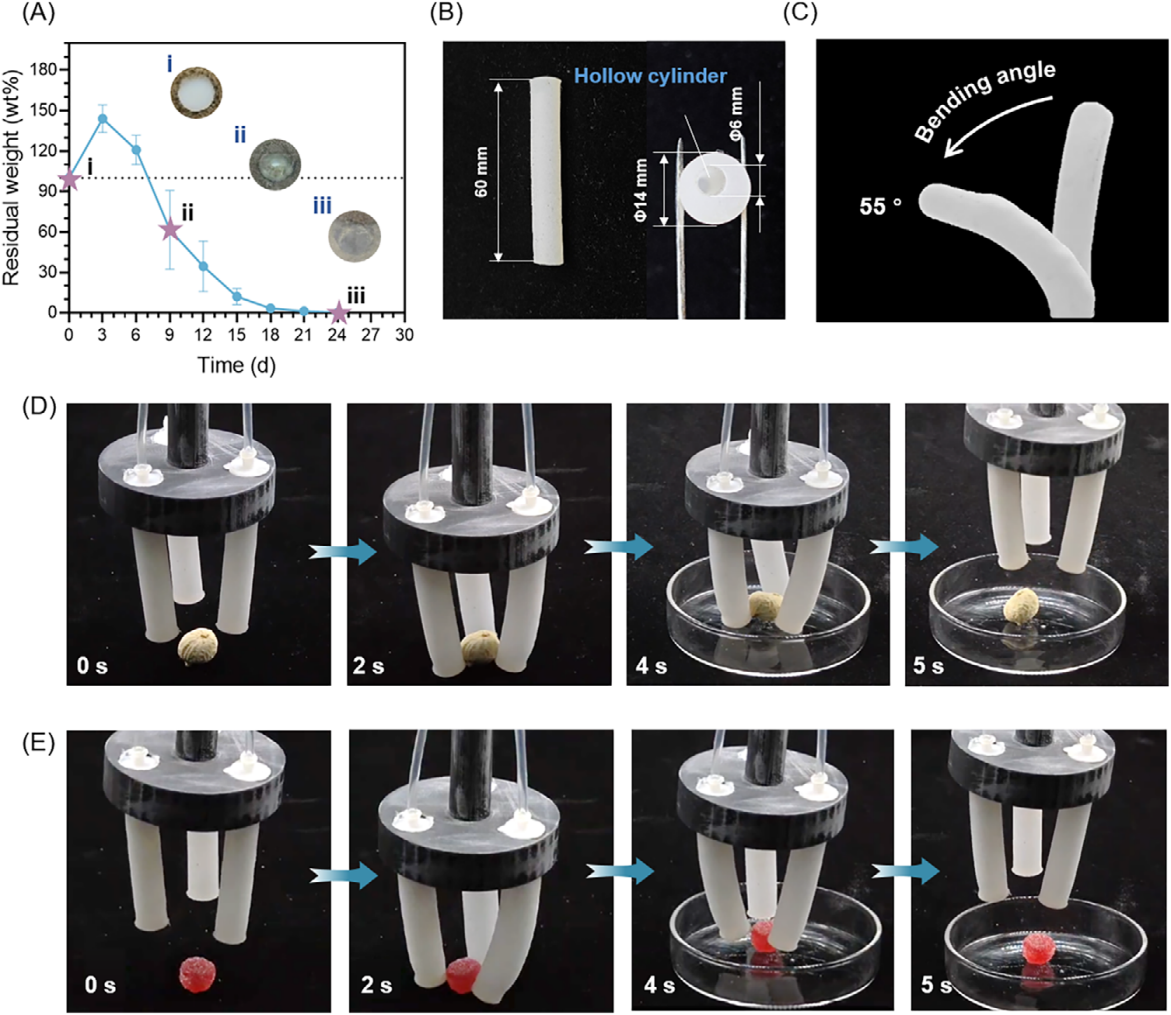
Edible and biodegradable starch hydrogel for soft robots. A) Degradation curve of starch hydrogel in soil. B) Eccentric‐cylindrical starch hydrogel fingers. C) The grab bending degree of the starch hydrogel. Actuations of the soft gripper for grabbing D) peanuts and E) elastic gummies.

To evaluate the environmental compatibility of starch hydrogels, a degradation test was conducted in biologically active soil (maintained at 20 °C, containing humic substances of decayed leaves). As depicted in **Figure** **5**A, the hydrogel initially displayed hydrophilic swelling during the first 3 days (mass increase: ≈44%), attributed to synergistic water absorption by starch polymers and glycerol. Subsequently, microbial colonization triggered and accelerated the degradation of hydrogel, culminating in its materials decomposing thoroughly within 24 days. The complete and rapid degradation profile of starch hydrogel highlighted the potential of this green material to mitigate persistent pollution from conventional polymer‐based electronic waste. Additionally, The starch hydrogel stored in air for 8 months maintained good mechanical properties (*σ_max_* = 36 kPa, *ε_max_* = 107.11%, G0.7/E0.6; Figure S9, Supporting Information), demonstrating its stability in air.

Based on the tunable mechanical properties of starch hydrogel (maximum tensile strength: 34–192 kPa; maximum strain: 192.30–361.39%), a pneumatically actuated soft gripper was engineered. The system featured three units (Figure S10, Supporting Information): i) a 3D‐printed ABS handle serving as the structural backbone; ii) a pneumatic driver modulating air pressure cycles (0‐65 kPa); and iii) eccentric‐cylindrical starch hydrogel fingers mimicking human grasping kinematics (Figure 5B; Movie S4, Supporting Information). Under pressurization, anisotropic bending (a rotation of 55 ± 2°) arose from the asymmetric strain distribution within the hydrogel matrix, enabling targeted object capture with good toughness and elasticity (Figure 5C; Movie S5, Supporting Information). The soft gripper demonstrated operational versatility by securely lifting irregular objects such as peanuts (length 18.4 ± 0.5 mm, width 12.0 ± 0.8 mm, height 11.0 ± 1.2 mm, weight 0.87 ± 0.15 g) and elastic gummies (length 13.5 ± 0.6 mm, width 13.5 ± 0.6 mm, height 12.4 ± 0.9 mm, weight 1.83 ± 0.20 g) via coordinated finger convergence (Figure 5D,E; Movies S6 and S7, Supporting Information). Three fingers bent to the center point under‐inflation to clamp the objects and released air to return the fingers to their original relaxed state. Overall, the starch hydrogel successfully combined edible and degradable properties with excellent mechanical performance, and had great potential to be used as soft materials in food, drug, and flexible robot fields.

## 3 Conclusion

This study applied the phase separation strategy of solvent‐antisolvent co‐modulation to tailor hyperelastic starch hydrogels, composed of starch (22.26‐54.42 wt%), bound water (20.81‐56.51 wt%), and glycerol (6.71‐39.84 wt%), achieving excellent structural and mechanical tunability. The ethanol phase (as antisolvent) drove the reorganization of short‐range starch chains into V/B‐type crystalline domains or blocks at micro‐nano scales, as the energy‐dissipative phase of local aggregation topography. The glycerol (as a solvent with water) facilitated the generation of bound water in the final starch hydrogel to form dynamic hydrogen‐bonding soft matrices. By manipulating the glycerol/ethanol ratio, we regulated the degrees of solvent‐system instability and starch affinity to control phase‐separation kinetics. Generally, the larger the glycerol/ethanol ratios, the greater the macropores in hydrogels formed, and the stronger the hydrogen bonding in the hydrogel spatial networks. Increasing the initial glycerol content reduced the maximum tensile stress and increased the maximum strain.

We also observed a significant dual synergistic effect (the hydrogel showed high maximum strain and stress) by increasing the initial ethanol content and maintaining a constant glycerol level. The Young's modulus of the hydrogel was negatively correlated with glycerol and positively with ethanol, which synergistically controlled network stiffness. Furthermore, the hydrogel was suitable for the application scenarios requiring cyclic load durability. The hyperelastic starch hydrogel with edible and degradable all‐components had excellent mechanical properties for pneumatic‐driven soft devices, showing great potential for the development of sustainable, green, and functional biomaterials.

## 4 Experimental Section

### Materials

Native corn starch powder (Hebei Yuxing Food Co., Ltd., China) was used as the main component of the as‐prepared hydrogel network. Dimethyl sulfoxide (DMSO)‐d6‐TMS (0.03%) and absolute ethanol were purchased from Sigma–Aldrich (USA) and used without further purification. Glycerol was purchased from Macklin (China).

### Fabrication of Hyperelastic Starch Hydrogel

The hydrogels were synthesized through a solvent displacement protocol. Corn starch (10 wt.%) was dispersed in a glycerol‐water solution (40–70%) and gelatinized in a 100 °C water bath for 30 min under continuous magnetic stirring (500 rpm). Then, a defined volume ratio of 0.6–1.2 v/v ethanol relative to glycerol/water solution (simplified as v_ethanol_/v_glycerol+water_), was added to the solution in a 50 °C water bath and kept stirring for 1 h. Subsequently, the solution was centrifuged at 4000 rpm for 10 min to remove most of the free water, glycerol, and ethanol. Finally, it was cross‐linked and regenerated in a 50 °C oven for 2 days to form a starch hydrogel. The samples were named according to the glycerol and ethanol volume ratio used. For example, G0.4/E0.6 was 40% of glycerol aqueous solution, and the amount of ethanol was 0.6 times that of glycerol aqueous solution. The initial content of materials for the as‐prepared hydrogel was shown in Table S1 (Supporting Information). A non‐ethanol control (G0.6/E0) was designed by gelatinizing starch in the glycerol‐water solution in a 100 °C water bath for 30 min, followed by 50 °C equilibration.

### Fabrication of the Soft Gripper

The starch hydrogel was first molded into a cylindrical shape (Φ10 mm, L = 100 mm), followed by being punctured with a mechanical drill into an eccentric cylindrical cavity (Φ5 mm, L = 90 mm) to act as a finger of the soft gripper. Three fingers of the same size were connected to the substrate printed with silica gel to form a soft gripper.

### Structural and Functional Characterizations—Cryo‐Electron Microscopy (Cryo‐EM)

The micro‐morphology of the starch hydrogel was characterized by Cyro‐EM (EM VCT500, Leica, Germany). The hydrogel sample was applied to the grids (Quantifoil 1.2/1.3). Grids were then plunge‐frozen in the freezer (Thermo Fisher, Vitrobot) with liquid nitrogen. The crystal water of the sample was sublimated. The sample was transferred to a cryo‐electron microscope and observed with a CCD camera (Thermo Fisher, Ceta).

### Structural and Functional Characterizations—Component Content

The sample was cut into pieces and placed in an 80 °C oven until the weight remained unchanged. The water content (*W_w_*), glycerol content (*W_g_*), and starch content (*W_s_*) of hydrogels were calculated using Equations (1), (2) and (3).

(1) $$\mathrm{The}\;\mathrm{water}\;\mathrm{content}=\frac{W_{b}-W_{a}}{W_{b}}\times \;100\%$$

(2) $$\mathrm{The}\;\mathrm{glycerol}\;\mathrm{content}=\frac{W_{b}-W_{a}-W_{s}}{W_{b}}\times \;100\%$$

(3) $$\mathrm{The}\;\mathrm{starch}\;\mathrm{content}=\frac{W_{s}}{W_{b}}\times \;100\%$$

where *W_b_* and *W_a_* are the weight of the hydrogel before and after drying in the oven, *W_s_* is the initial amount of starch added.

### Structural and Functional Characterizations—Low‐Field Nuclear Magnetic Resonance (LF‐NMR)

The hydration state of hydrogels was performed using an LF‐NMR spectrometer (NMI20‐015V‐I, Niumag, China) operating at a 0.5 T magnetic field strength; the relaxation times of hydrogels were calculated under precise temperature control (32.0 ± 0.1 °C) and a proton resonance frequency of 21.16 MHz.

### Structural and Functional Characterizations—X‐Ray Powder Diffraction (XRD)

The crystalline properties were characterized using an X‐ray diffractometer (Bruker D8 Advance, Germany) equipped with a monochromated Cu Kα radiation source (λ = 0.1542 nm). Measurement parameters were systematically configured as follows: angular range from 4° to 40° (2θ), scan speed maintained at 10°/min, under optimized excitation conditions of 40 kV accelerating voltage and 50 mA filament current.

### Structural and Functional Characterizations—Fourier Transform Infrared Spectrometer (FTIR)

The molecular structure of the hydrogel was characterized using FTIR (Nicolet iS50, Thermo Fisher Scientific, USA) in attenuated total reflectance (ATR) mode. The spectral acquisition involved 32 co‐added scans with 4 cm^‐^¹ resolution across the mid‐infrared region (4000–400 cm^‐^¹). Spectral deconvolution and quantitative absorbance ratio analysis were executed using the OMNIC 8.0 software package.

### 1H Nuclear Magnetic Resonance (1H NMR)

Nuclear magnetic resonance characterization was performed using a spectrometer (AVANCE III HD 600 MHz, Bruker, Germany). The hydrogel was ground into powder in liquid nitrogen and dissolved in deuterated dimethyl sulfoxide (DMSO‐d6) containing 0.03% tetramethylsilane (TMS) as an internal reference. 1H NMR spectra were acquired with the following parameters: spectral width 12.02 kHz, relaxation delay 1.0 s, receiver gain 183, and 512 accumulated scans.

### 1H Nuclear Magnetic Resonance (1H NMR)—Dynamic Mechanical Analysis (DMA)

DMA tests were performed with an RSA‐G2 analyzer (Water Corporation, USA). Dynamic frequency sweeps were performed at angular velocities ranging from 0.01 to 100 Hz. All rheological measurements were performed with a vibration strain of 1% and at 25 °C using parallel plate geometry (width 5 mm, length 20 mm, thickness 2 mm).

### 1H Nuclear Magnetic Resonance (1H NMR)—Tensile Testing

The hydrogels were cut into dog‐bone‐shaped specimens with a gauge width of 2 mm for regular tensile testing. The single stress–strain curves were obtained by a mechanical tester (HF‐9001, Ligao, China) equipped with 100‐N loading cells. Resilience property was obtained by another tester (Instron 5944, Instron Corporation, USA) with a strain rate of 0.5 mm mm^−1^. Ten tests were conducted for each data point.

### 1H Nuclear Magnetic Resonance (1H NMR)—Soil Burial Biodegradation Assessment

The hydrogel sample was buried in the soil, which was rich in microorganisms. The soil was taken from the natural environment and placed in an incubator with a temperature of 30 °C and a relative humidity of 30%. The residual weight of the sample after microbial degradation was recorded every 2 days.

### Statistical Analysis

All experiments were performed independently, with each condition replicated at least three times. Results were presented as means ± standard deviation (SD). Statistical analyses were conducted using either GraphPad Prism 8 or Origin 2024 software.

## Conflict of Interest

The authors declare no conflict of interest.

## Author Contributions

S.Y. and H.H. contributed equally to this work. S.Y. contributed to data curation, methodology, validation, investigation, writing the original draft, and conceptualization. H.H. was responsible for data curation, methodology, software, and writing the original draft. M.Z. handled data curation, methodology, and validation. Q.Z. was focused on data curation and methodology. D.L. and S.Q. provided supervision, writing, reviewing, and editing. G.M. and E.X. oversaw funding acquisition, writing, reviewing, editing, methodology, conceptualization, and supervision.

## Supporting information



Supporting Information

Supplemental Movie 1

Supplemental Movie 2

Supplemental Movie 3

Supplemental Movie 4

Supplemental Movie 5

Supplemental Movie 6

Supplemental Movie 7

## Data Availability

The datasets generated during and/or analyzed during the current study are available from the corresponding author on reasonable request.
