# Supplementary material for: Hyperelastic Starch Hydrogel Configures Edible and Biodegradable All‐Components for Soft Robots
Source: Adv Sci (Weinh). 2025 Jul 7;13(15):e07216. doi: 10.1002/advs.202507216 (PMC13042779; doi:10.1002/advs.202507216)
Supplement: Supplementary file 1 — Supporting Information [file ADVS-13-e07216-s001.docx]

Supporting Information

Hyperelastic Starch Hydrogel Configures Edible and Biodegradable All-Components for Soft Robots

Siyu Yao^1#^, Haohao Hu^1,3#^, Mengfan Zhang^2^, Qingqing Zhu^1,3^, Donghong Liu^1,3^, Shaoxing Qu^2^, Guoyong Mao^2^*, Enbo Xu^1,3^*

*^1^College of Biosystems Engineering and Food Science, National Engineering Laboratory of Intelligent Food Technology and Equipment, Zhejiang Key Laboratory for Agro-Food Processing, Fuli Institute of Food Science, Zhejiang University, Zhejiang University, Hangzhou 310058, China*

*^2^ Department of Engineering Mechanics, Zhejiang University, Hangzhou 310027, China*

*^3^Innovation Center of Yangtze River Delta, Zhejiang University, Jiaxing 314102, China*

** Corresponding author:*

*E-mail address: guoyongmao@zju.edu.cn (G. Mao); enboxu@zju.edu.cn (E. Xu).*

**Table S1.** Experimental design used for the preparation of starch hydrogels.

| Sample | Starch (g) | Glycerol (mL) | Water (mL) | Ethanol (mL) |
| --- | --- | --- | --- | --- |
| G0.4/E0.6 | 10 | 80 | 120 | 120 |
| G0.4/E0.8 | 10 | 80 | 120 | 160 |
| G0.4/E1.0 | 10 | 80 | 120 | 200 |
| G0.4/E1.2 | 10 | 80 | 120 | 240 |
| G0.5/E0.6 | 10 | 100 | 100 | 120 |
| G0.5/E0.8 | 10 | 100 | 100 | 160 |
| G0.5/E1.0 | 10 | 100 | 100 | 200 |
| G0.5/E1.2 | 10 | 100 | 100 | 240 |
| G0.6/E0.6 | 10 | 120 | 80 | 120 |
| G0.6/E0.8 | 10 | 120 | 80 | 160 |
| G0.6/E1.0 | 10 | 120 | 80 | 200 |
| G0.6/E1.2 | 10 | 120 | 80 | 240 |
| G0.7/E0.6 | 10 | 140 | 60 | 120 |
| G0.7/E0.8 | 10 | 140 | 60 | 160 |
| G0.7/E1.0 | 10 | 140 | 60 | 200 |
| G0.7/E1.2 | 10 | 140 | 60 | 240 |

**Table S2.** Relative composition of starch, glycerol and water in as-prepared hydrogels.

| Sample | Starch (wt%) | Glycerol (wt%) | Water (wt%) |
| --- | --- | --- | --- |
| G0.4/E0.6 | 29.35 | 31.60 | 39.05 |
| G0.4/E0.8 | 40.15 | 30.03 | 29.82 |
| G0.4/E1.0 | 48.63 | 27.22 | 24.15 |
| G0.4/E1.2 | 54.42 | 24.77 | 20.81 |
| G0.5/E0.6 | 25.68 | 35.81 | 38.51 |
| G0.5/E0.8 | 37.45 | 29.58 | 32.97 |
| G0.5/E1.0 | 44.15 | 27.66 | 28.19 |
| G0.5/E1.2 | 50.05 | 23.18 | 26.77 |
| G0.6/E0.6 | 23.46 | 39.84 | 36.70 |
| G0.6/E0.8 | 31.68 | 17.62 | 50.70 |
| G0.6/E1.0 | 36.78 | 6.72 | 56.50 |
| G0.6/E1.2 | 37.76 | 9.89 | 52.35 |
| G0.7/E0.6 | 22.26 | 37.95 | 39.79 |
| G0.7/E0.8 | 28.97 | 28.55 | 42.48 |
| G0.7/E1.0 | 33.04 | 13.34 | 53.62 |
| G0.7/E1.2 | 37.02 | 8.82 | 54.16 |

**Table S3.** Comparing mechanical properties of hydrogels with varying starch contents.

| Hydrogel | Starch content (wt%) | Strain (%) | Ref |
| --- | --- | --- | --- |
| Carboxymethyl starch/polyacrylamide/ferric ion | ~0.71 | 1400-1600 | [1] |
| Starch/poly(vinyl alcohol) | ~1.02 | - | [2] |
| Starch/polyacrylamide | ~1.93 | - | [3] |
| Starch/polyacrylamide/phytic acid | ~6.43 | 30k-40k | [4] |
| Starch/ calcium chloride /glycerol | ~13.51 | 150-180 | [5] |
| Starch/polyacrylic acid | ~19.31 | - | [6] |
| Starch/poly(vinyl alcohol)/borax/carbon nanotube | ~38.76 | ~10k | [7] |
| Starch/glycerol | 54.42 | 300-400 | this work |

**Table S4.** Mechanical properties of hyperelastic starch hydrogels.

| Sample | Maximum strain (*ε*_max_) / % | Maximum tensile stress (*σ*_max_) / kPa | Young’s modulus (E) /kPa |
| --- | --- | --- | --- |
| G0.4/E0.6 | 328.501 | 44 | 36 |
| G0.4/E0.8 | 261.793 | 131 | 133.2 |
| G0.4/E1.0 | 192.305 | 131 | 158.4 |
| G0.4/E1.2 | 252.127 | 192 | 205.8 |
| G0.5/E0.6 | 252.293 | 55 | 52.8 |
| G0.5/E0.8 | 227.339 | 78 | 90.0 |
| G0.5/E1.0 | 263.037 | 104 | 104.1 |
| G0.5/E1.2 | 358.288 | 144 | 117 |
| G0.6/E0.6 | 281.136 | 62 | 56.1 |
| G0.6/E0.8 | 330.161 | 72 | 60.9 |
| G0.6/E1.0 | 262.291 | 70 | 67.2 |
| G0.6/E1.2 | 327.796 | 85 | 70.2 |
| G0.7/E0.6 | 194.359 | 34 | 40.8 |
| G0.7/E0.8 | 226.800 | 82 | 89.1 |
| G0.7/E1.0 | 228.252 | 54 | 63.3 |
| G0.7/E1.2 | 361.399 | 75 | 60.9 |


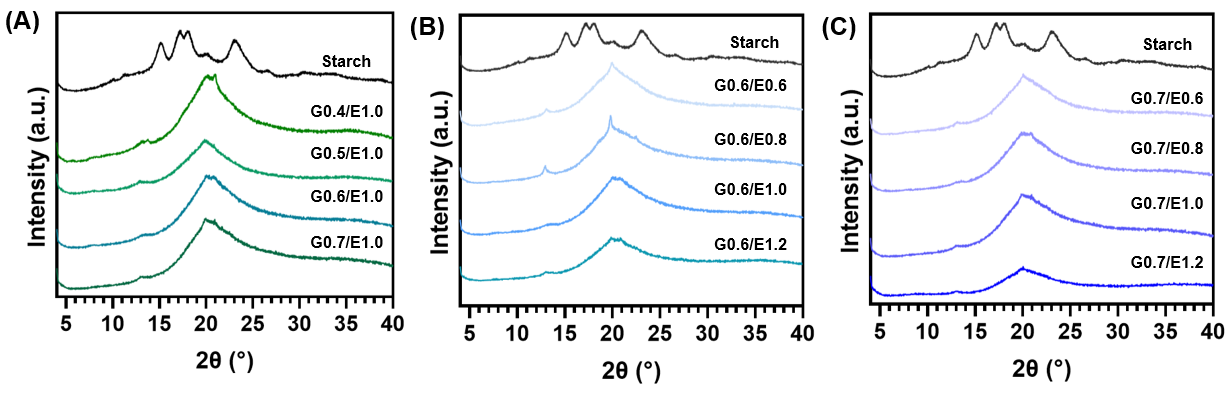


**Figure S1**. Crystalline properties of starch hydrogels in XRD patterns. Samples include G0.4/E1.0, G0.5/E1.0, G0.6/E1.0, G0.7/E1.0, G0.6/E0.6, G0.6/E0.8, G0.6/E1.2, G0.7/E0.6, G0.7/E0.8, G0.7/E1.2.


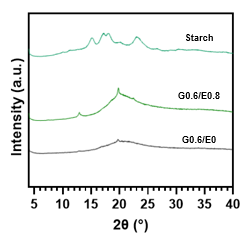


**Figure S2**. Effect of ethanol on the crystallinity of hydrogel. G0.6/E0, the control group without ethanol action.


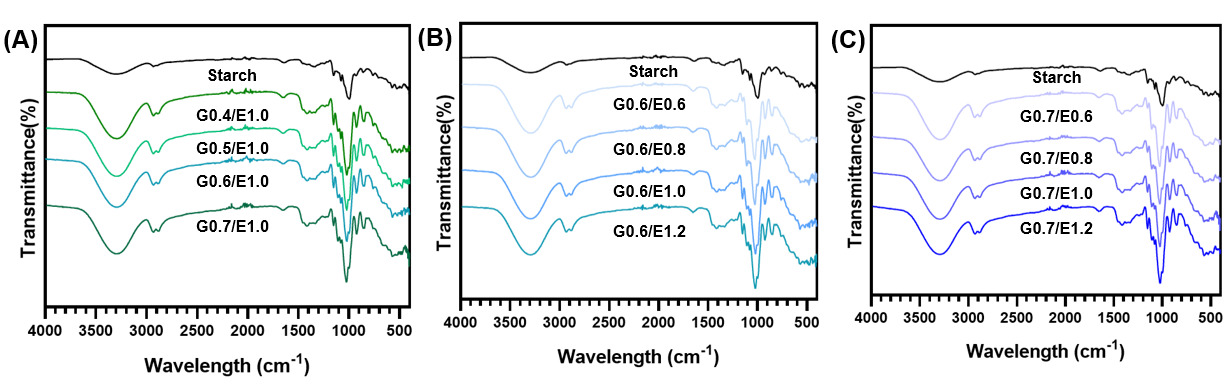


**Figure S3**. FTIR spectra of starch hydrogels, i.e., G0.4/E1.0, G0.5/E1.0, G0.6/E1.0, G0.7/E1.0, G0.6/E0.6, G0.6/E0.8, G0.6/E1.2, G0.7/E0.6, G0.7/E0.8, G0.7/E1.2.


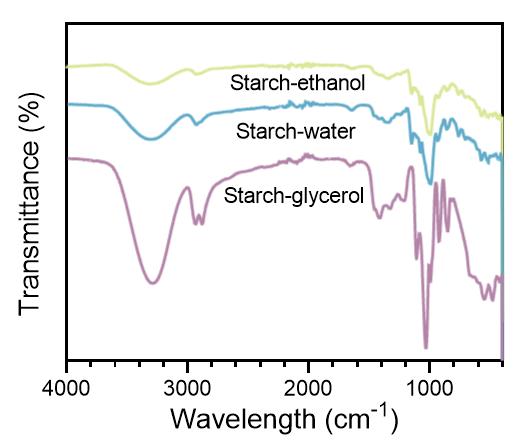


**Figure S4**. FTIR spectra of starch-ethanol, starch-water and starch-glycerol.

Control groups of starch-water, starch-glycerol, and starch-ethanol were prepared as follows. Starch was suspended in water, glycerol, or ethanol to form 3 wt% suspensions, gelatinized at 100°C, then oven-dried at 50°C for 2 d to remove free water. FTIR analysis revealed that the absorption peak intensity at ~3200 cm⁻¹ followed the order: starch-glycerol > starch-water > starch-ethanol. This indicates the starch affinity strength order: glycerol > water > ethanol.


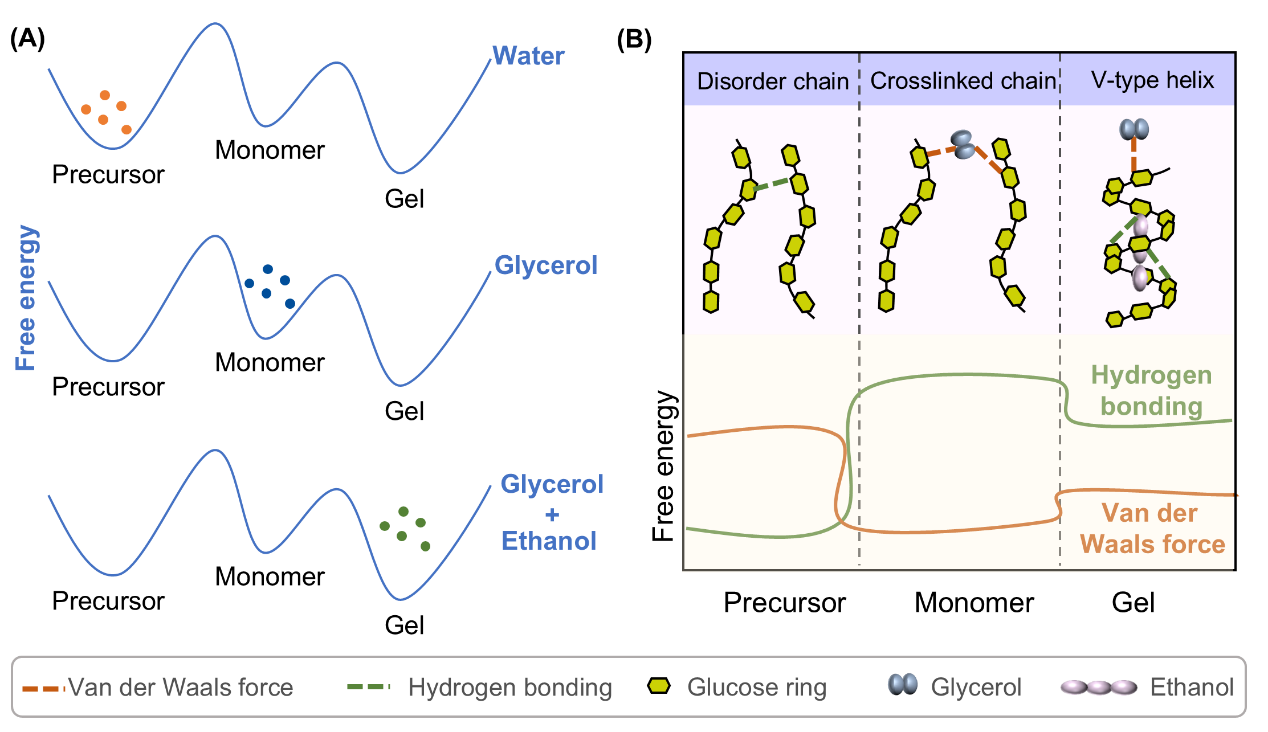


**Figure S5.** Schematic representation of free energy landscape for starch self-assembly using different solvent triggers. (A) Glycerol and ethanol change the shape of the free energy landscape and relative stability of the monomer and gel states. (B) conformations change with competing forces of hydrogen bonding and van der Waals force.


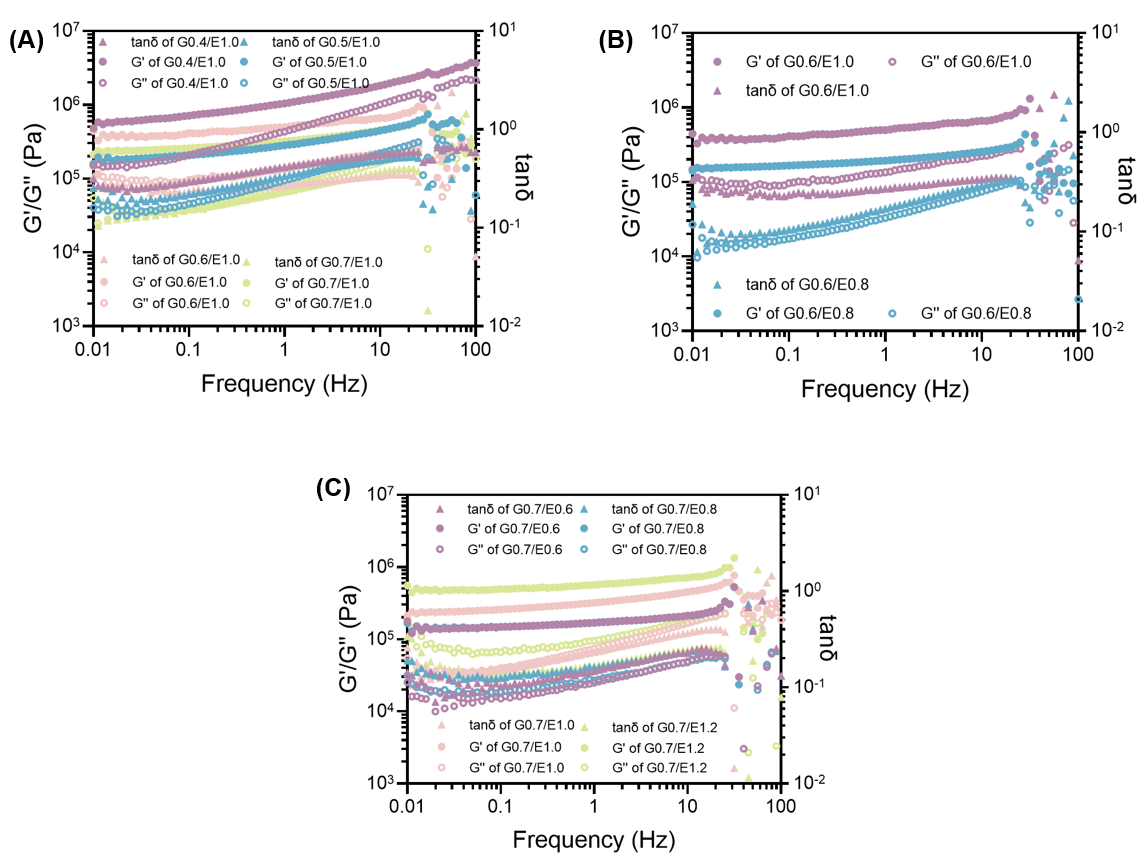


**Figure S6**. The frequency sweep of starch hydrogels. (A) G0.4/E1.0, G0.5/E1.0, G0.6/E1.0, G0.7/E1.0. (B) G0.6/E1.0, G0.6/E0.8. (C) G0.7/E0.6, G0.7/E0.8, G0.7/E1.0, G0.7/E1.2.


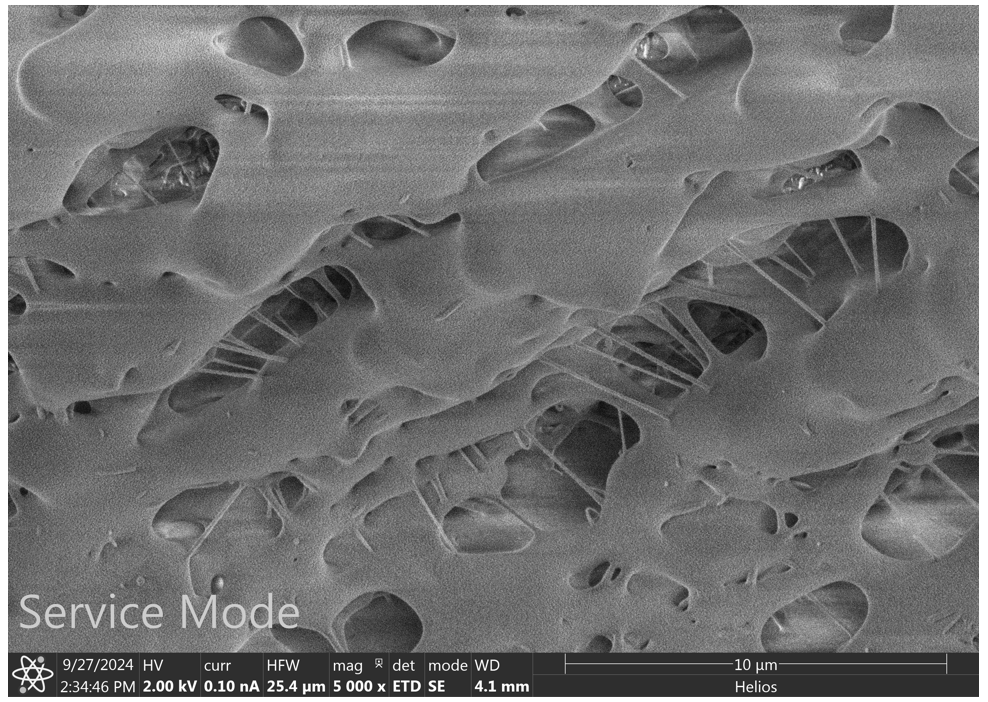


**Figure S7**. Cryo-EM images of as-prepared starch hydrogel of G0.7/E1.2.


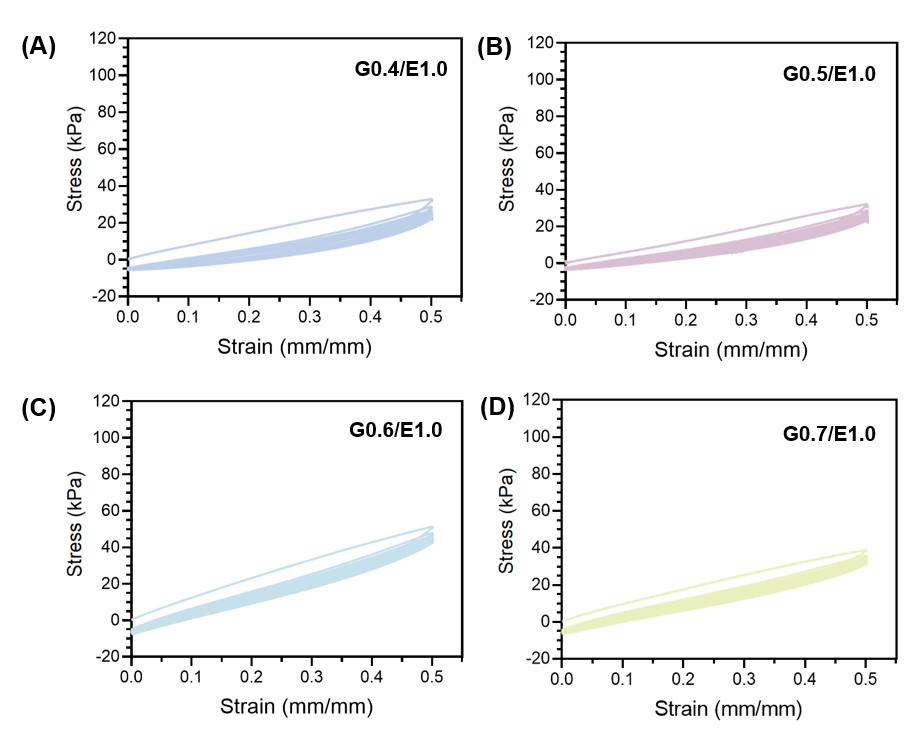


**Figure S8**. Cyclic loading of starch hydrogels, i.e., G0.4/E1.0, G0.5/E1.0, G0.6/E1.0 and G0.7/E1.0.


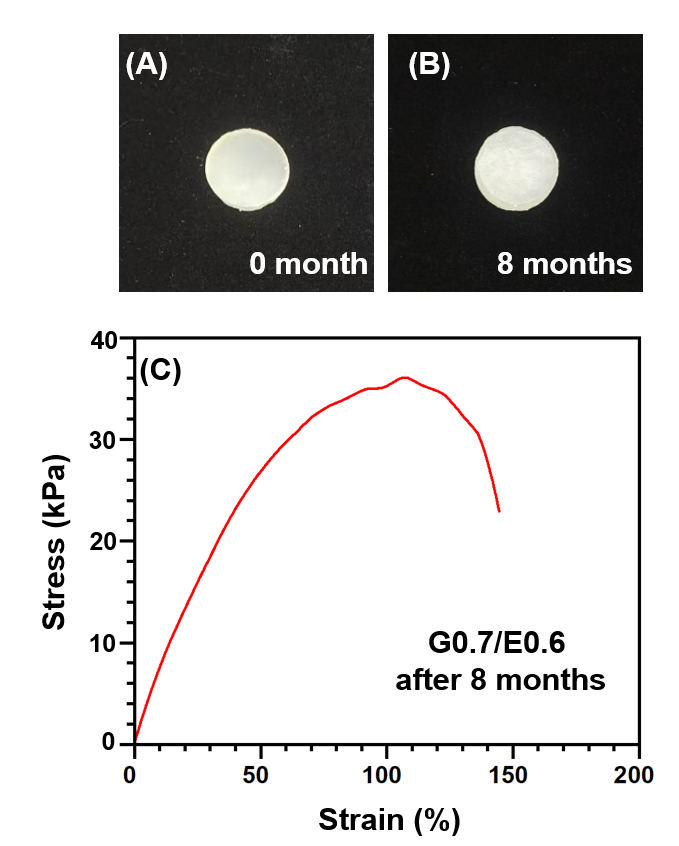


**Figure S9.** Changes in G0.7/E0.6 appearance and mechanical properties after 8 months. (A) Appearance changes after 8 months. (B) Mechanical performance of hyperelastic starch hydrogel after 8 months.


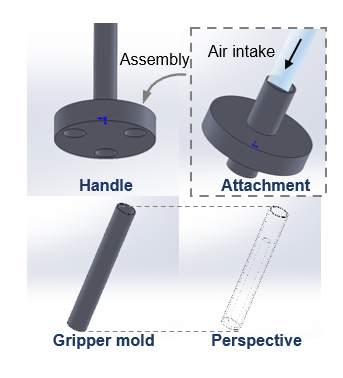


**Figure S10**. Component of pneumatically actuated soft gripper

**Reference:**

[1] S. Liu, X. Wang, Y. Peng, Z. Wang, R. Ran, Highly stretchable, strain-sensitive, and antifreezing macromolecular microsphere composite starch-based hydrogel, *Macromolecular Materials and Engineering* 2021, 306 (9), 2100198.

[2] P. Sankarganesh, Parthasarathy, A. G. Kumar, S. Ragu, M. Saraniya, N. Udayakumari, Preparation of PVA/starch hydrogel and its in-vitro drug release potential against pus-inducing pathogenic strain and breast cancer cell line, *Journal of Sol-Gel Science and Technology* 2022, 101 (3), 571.

[3] Q. Qin, Q. Tang, B. He, H. Chen, S. Yuan, X. Wang, Enhanced proton conductivity from phosphoric acid-incorporated 3D polyacrylamide-graft-starch hydrogel materials for high-temperature proton exchange membranes, *Journal of Applied Polymer Science* 2014, 131 (16), 164972.

[4] J. Zhao, R. Chen, D. Cheng, X. Yang, H. Zhang, J. Zheng, R. Hu, Extremely ultrahigh stretchable starch-based hydrogels with continuous hydrogen bonding, *Advanced Functional Materials* 2025, 35 (8), 2415530.

[5] C. Ma, F. Xie, L. Wei, C. Zheng, X. Liu, L. Wang, P. Liu, All-starch-based hydrogel for flexible electronics: strain-sensitive batteries and self-powered sensors, *ACS Sustainable Chemistry & Engineering* 2022, 10 (20), 6724.

[6] Y.-N. Zhang, J.-Y. Cui, S.-A. Xu, Effects of chain structures of corn starches on starch-based superabsorbent polymers, *Starch - Stärke* 2015, 67 (11-12), 949.

[7] Z. Gu, X. Chen, L. Xu, X. Wang, Z. Lin, T. Chen, A Simple physical kneading strategy to prepare an ultrahigh-stretchable conductive starch/poly(vinyl alcohol)/borax/carbon nanotube hydrogel for flexible capacitive electronics, *ACS Applied Polymer Material*s 2025, 7 (3), 1448.
